# The CRISPR genotypes and genetic diversity of different serogroups of nontyphoidal *Salmonella* in Guizhou Province, 2013–2018

**DOI:** 10.1371/journal.pone.0278321

**Published:** 2022-12-15

**Authors:** Guihuan Bai, Lv You, Li Long, Dan Wang, Ming Wang, Junhua Wang, Jianhua Li, Xiaoyu Wei, Shijun Li

**Affiliations:** 1 The Laboratory of Bacterial Disease, Experimental Center, Guizhou Provincial Center for Disease Control and Prevention, Guiyang, People’s Republic of China; 2 The Laboratory of Bacterial Disease, Tongren City Center for Disease Control and Prevention, Tongren, People’s Republic of China; 3 Institute of Communicable Disease Control and Prevention, Guizhou Provincial Center for Disease Control and Prevention, Guiyang, People’s Republic of China; 4 School of Public Health, the Key Laboratory of Environmental Pollution Monitoring and Disease Control, Ministry of Education, Guizhou Medical University, Guiyang, People’s Republic of China; Adamas University, INDIA

## Abstract

Nontyphoidal *Salmonella* is a bacterial and foodborne pathogen that poses a severe public health threat. However, the genetic diversity of different serogroups of nontyphoidal *Salmonella* in Guizhou is unknown. This study aimed to obtain the RNA secondary structure of the typical direct repeat sequences, the characteristics of clustered regularly interspaced short palindromic repeats (CRISPR) genotypes, and the genetic diversity of different serogroups of nontyphoidal *Salmonella* strains. The 342 nontyphoidal *Salmonella* strains were collected from nine cities (prefectures) of Guizhou province during 2013–2018, serotyped by slide agglutination, and examined the molecular genotypes by CRISPR method. The strains were divided into five serogroups. The dominant serogroup was group B (47.08%), followed by group D1 (36.55%). One hundred and thirty-five CRISPR genotypes were detected with 108 novel spacer sequences amongst 981 unique spacer sequences. The diversity of nontyphoidal *Salmonella* CRISPR loci was not only the deletion, duplication, or point mutation of spacer sequences but also the acquisition of new spacer sequences to form novel genotypes. The CRISPR genotyping was an effective typing method that could reveal the genetic diversity of different nontyphoidal *Salmonella* serotypes except for *S*. Enteritidis.

## Introduction

Nontyphoidal *Salmonella*, a gram-negative bacillus, can cause salmonellosis through contaminated water, food, and feces and is the third leading cause of human deaths from diarrheal diseases worldwide [1]. In 2017, the European Food Safety Authority (EFSA) reported nontyphoidal *Salmonella* as the most common cause of foodborne illness outbreaks [2]. In recent years, bacterial food poisoning caused by nontyphoidal *Salmonella* ranked first among poisoning incidents in China [3]. Currently, nontyphoidal *Salmonella* can be divided into more than 50 serogroups, of which serogroup C (C1 and C2) is the most common serogroup in the United States (25.7%), followed by serogroup B (20.5%) [4]. However, serogroup B is the most common serogroup of nontyphoidal *Salmonella* in China, accounting for 56.9% [5]. The surveillance of foodborne disease outbreaks in Guizhou province showed nontyphoidal *Salmonella* infection ranked among the top three bacterial foodborne outbreaks from 2011 to 2016 [6]. The report also indicated that nontyphoidal *Salmonella* was the top pathogenic bacteria detected from 2015 to 2017 in Guizhou province [7]. In addition, the predominant serotype of foodborne nontyphoidal *Salmonella* was serogroup B (41.66%), followed by serogroup C2 (16.67%) [8]. Therefore, we should pay enough attention to nontyphoidal *Salmonella* infection, strengthen the monitoring of foodborne diseases caused by nontyphoidal *Salmonella*, and further control the spread of pathogens. Until now, approximately 1586 serotypes of *Salmonella enterica subspecies* were found, of which about 99% of serotypes may cause human or animal infection [9]. Traditional serological typing methods are laborious and time-consuming due to the complexity of nontyphoidal *Salmonella* serotypes, which cannot explain the epidemiological relationship between the same serotypes. However, it is critical to type and trace the source of nontyphoidal *Salmonella* outbreak quickly. Molecular typing techniques such as pulsed-field gel electrophoresis (PFGE), multilocus sequence typing (MLST), and multiple-locus variable-number tandem-repeat analysis (MLVA) have been used to track the origins of bacterial diseases. PFGE was regarded as the "gold standard" for bacterial molecular types and was used to trace the source of a pathogenic bacteria infection from various sources [10]. However, the PFGE molecular typing method is complex and less effective in exploring the genetic relationship of the same serotype of nontyphoidal *Salmonella* [11]. Due to the conservative nature of MLST housekeeping genes, it is also impossible to classify some highly homologous pathogens [12,13]. Compared with PFGE and MLST, the Clustered Regularly Interspaced Short Palindromic Repeats (CRISPR) are more suitable and simplified for distinguishing the outbreak strains of nontyphoidal *Salmonella*. Moreover, it corresponds well to nontyphoidal *Salmonella* serotypes and can reveal the genetic diversity and evolutionary relationships of strains from different sources [14–16].

CRISPR is an acquired and heritable biological immune defense system formed by RNA-mediated interference with exogenous nucleic acid fragments, consisting of conserved direct repeats (DRs) of 22–47 bp and spacer sequences of 21–72 bp. Moreover, CRISPR is one of the elements with the fastest genetic evolution in the bacterial genome and widely distributed in bacterial and archaeal genomes [17]. The DRs are usually conserved and have palindromic structures, which can be transcribed to form RNA secondary structures [18]. The diversity of spacers can provide a reliable basis for bacterial identification and evolutionary analysis, which can be used as a target for typing [19]. *Salmonella* usually carries two CRISPR loci, CRISPR1 and CRISPR2. Different *Salmonella* strains of the same serotype can be distinguished by analyzing the spacer sequences of CRISPR1 and CRISPR2 loci [20]. The utility of this subtyping method in surveillance and outbreak detection has been already demonstrated in several *Salmonella* serotypes in Québec, China, and France [19–21]. This indicates that CRISPR profiles can be useful as a complementary approach to determine source attribution in foodborne outbreaks. CRISPR analysis was also able to distinguish outbreak strains from epidemiologically unrelated isolates [21].

In this study, we collected 342 nontyphoidal *Salmonella* strains from humans to understand the RNA secondary structure of DRs, genotypes, and genetic diversity by CRISPR typing in Guizhou province.

## Materials and methods

### Ethics statement

The present study was reviewed and approved by the Ethics Review Committee of Guizhou Provincial Center for Disease Control and Prevention. All data/isolates were analyzed anonymously.

### Bacterial isolation and identification

Based on the routine surveillance of infectious diarrhea in Guizhou Province, stool samples were collected from patients with three or more consecutive diarrhea and abnormal stools (loose stools, watery stools, mucus stools, pus, bloody stools) within 24 hours. The stool samples had been enriched *Salmonella* bacteria in SBG broth (Youkang, Beijing) for 18 hours, then inoculated to *Salmonella* chromogenic medium (CHROM Agar, France) for cultivation.The systematic biochemical method with API20E (BioMerieux, France) was used to identify nontyphoidal *Salmonella*. According to the Kauffmann and White scheme, the serotypes of nontyphoidal *Salmonella* were further performed by O and H antiserum (SSI, Denmark) agglutination.

### Bacterial DNA extraction

The bacterial suspension was made and subjected to a metal bath at 100°C for 10 minutes, then centrifuged at 13000 r/min for 10 minutes. The supernatant was bacterial DNA.

### PCR amplification and sequencing of CRISPR loci

The primers used to amplify the CRISPR1 and CRISPR2 loci of *Salmonella* were designed as previously described [19,22]. The PCR amplification of CRISPR loci was performed with 50 μl and contained 2 μl of genomic DNA (10 μmol/L), 2 μl of primers (10 μmol/ L), 25 μl of 2×Rapid Premix Taq (TakaRa, China), then 50 μl of pure water were added on top. The PCR reaction conditions were as follows: initial denaturation at 95°C for 2 min; 30 cycles of denaturation at 95°C for 30 s, annealing at 64°C for 30 s, annealing at 72°C for 90 s, and a final extension at 72°C for 10 min. PCR products (5μl) were subjected to electrophoresis at 1% agarose gel. Then, the positive products were sent to the company (Tianyihuiyuan, Beijing) for sequencing.

### Sequence analysis of CRISPR loci

The sequences of CRISPR1 and CRISPR2 loci were submitted to the CRISPRCasFinder website (https://crisprcas.i2bc.paris-saclay.fr/CrisprCasFinder/Index). The DRs and spacer arrangements were shown in the result of the website, sorted, and saved in Excel 2010. Furthermore, the typical DRs with the highest frequency of CRISPR loci were selected, and the RNA secondary structures were predicted by RNA fold (http://rna.tbi.univie.ac.at/cgi-bin/RNAWebSuite/RNAfold.cgi). At the same time, the minimum free energy (MFE, kcal/mol) was recorded. The regulation and criteria for the nomenclature of the spacer sequences were the same as those previously described for the different serotypes [19]. For the new spacer sequence not reported in the spacer database, we named them Unknown1, Unknown2, and Unknown3. The CRISPR genotypes were generated by combining the CRISPR1 and CRISPR2 loci into a single allele, and spacer sequences were shown for each strain. Newly discovered CRISPR genotypes were indicated by the prefix "New" and the various serotypes were identified by various numeric suffixes. Additionally, for the unknown serotypes of nontyphoidal *Salmonella*, newly discovered CRISPR genotypes were indicated by newtype1 and newtype2. Similarity analysis and genetic evolutionary relationships between strains of the same serogroup were performed using the unweighted pair group method (UPGMA) of BioNumerics 8.0 software.

## Results

### Strain identification and serogroup

From January 2013 to December 2018, a total of 342 nontyphoidal *Salmonella* strains were collected and isolated from nine cities and prefectures in Guizhou province. Among them, 88 strains were from Tongren city, 73 strains from Guiyang city, 60 strains from Zunyi city, 53 strains from Anshun city, 11 strains from Liupanshui city, 21 strains from Qiandongnan prefecture, 17 strains from Qiannan prefecture, ten strains from Qianxinan prefecture and nine strains from Bijie city. These strains were identified as nontyphoidal *Salmonella* by systematic biochemical method and divided into five serogroups: serogroup B, C1, C2-C3, D1, and E1 (Table 1). Serogroup B (47.08%, 161/342) was the predominant serogroup, followed by serogroup D1 (36.55%, 125/342). Twenty-four serotypes were identified among the 331 nontyphoidal *Salmonella* strains, of which *S*. Enteritidis was the most common serotype (37.46%, 124/331), followed by *S*. Typhimurium monophasic variant (21.45%, 71/331) and *S*. Typhimurium (17.82%, 59/331). The serotypes of 11 strains (3.22%, 11/342) were unknown.

**Table 1:** Numbers of CRISPR alleles and genotypes of 342 nontyphoidal *Salmonella* strains with different serotypes.

Serotypes	Strains	Ratio (%)	NO. of CRISPR alleles		CRISPR genotypes
			CRISPR1	CRISPR2	
*Salmonella* in group B	161	47.08	50	34	78
Typhimurium	59	17.25	29	21	TST1-53
1,4,[5],12:i	71	20.76			
Derby	13	3.80	12	3	DST1-13
Stanley	5	1.46	4	5	NewSST1、SST1-T4
Agona	9	2.63	1	2	AgST1-3
Saintpaul	2	0.58	2	1	SaST1-2
Hato	1	0.29	1	1	NewHaST1
Unidentified *Salmonella* in group B (SM201628)	1	0.29	1	1	Type1
*Salmonella* in group C1	14	4.09	9	12	14
Infant	5	1.46	3	4	IST1-IST5
Rissen	2	0.58		2	RiST1-2
Thompson	2	0.58	1	2	ThST1-2
Schwabach	2	0.58	2	2	ScST1-2
Singapore	1	0.29	1	1	SiST1
Concord	1	0.29	1		CoST1
Unidentified *Salmonella* in group C1 (SM201634)	1	0.29	1	1	Type5
*Salmonella* in group C2-C3	12	3.51	6	10	11
Kentucky	3	0.88	1	3	KeST1-3
Bovismorbifican	2	0.58	2	2	BoST1-2
Corvallis	2	0.58	1	1	CoST1
Hadar	2	0.58	1	1	HaST1-2
Tshiongwe	1	0.29		1	TsST1
Goldcoast	1	0.29	1	1	GoST1
Unidentified *Salmonella* in group C2-C3 (SM201426)	1	0.29		1	Newtype5
*Salmonella* in group D1	125	36.55	5	8	13
Enteritidis	124	36.26	5	8	EST1-EST13
Gallinarum	1	0.29	1	1	EST5
*Salmonella* in group E1	30	8.77	13	10	19
London	20	5.85	6	2	LST1-LST10
Meleagridis	1	0.29	1	1	NewHaST1
Amsterdam Ⅱ	1	0.29		1	AmST1
Unidentified *Salmonella* in group E1 (SM201316)	1	0.29	1	1	Newtype1
Unidentified *Salmonella* in group E1 (SM201317)	1	0.29	1		Type2
Unidentified *Salmonella* in group E1 (SM201318、SM201319)	2	0.58	1	1	Newtype2
Unidentified *Salmonella* in group E1 (SM201423)	1	0.29	1	1	Newtype3
Unidentified *Salmonella* in group E1 (SM201425)	1	0.29		1	Type3
Unidentified *Salmonella* in group E1 (SM201412)	1	0.29	1	1	Type4
Unidentified *Salmonella* in group E1 (SM201429)	1	0.29	1	1	Newtype4
Total	342	100.00			

### The CRISPR loci detection

In this study, 282 of the 342 nontyphoidal *Salmonella* strains were detected CRISPR1 and CRISPR2 loci, the other 32 strains were only detected the CRISPR1 locus, and 28 strains were only detected the CRISPR2 locus. Nine hundred eighty-one unique spacer sequences were identified in the 342 strains (Table 1), of which 108 spacer sequences were newly identified in the spacer sequence database, named Unknown1-108 (S1 Table). Three hundred and fourty-two nontyphoidal *Salmonella* strains were divided into 135 CRISPR genotypes.

### RNA secondary structure prediction of the typical DRs

**Fig 1:**
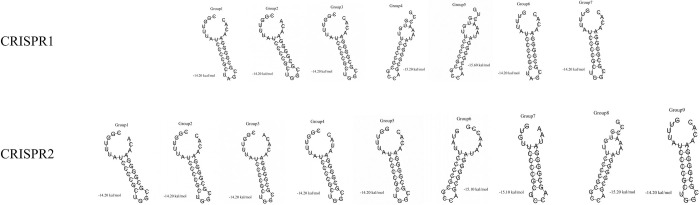
RNA secondary structures of typical DRs of CRISPR1 and CRISPR2 loci. The CRISPR1 loci were divided into seven groups, and the CRISPR2 loci were divided into nine groups.

The 342 nontyphoidal *Salmonella* strains contained a total of 8146 DRs, of which CRISPR1 loci contained 3685 DRs and CRISPR2 loci contained 4461 DRs. The typical DRs of CRISPR1 and CRISPR2 loci were divided into 16 groups, including seven groups of the CRISPR1 loci and nine groups of the CRISPR2 loci. RNA secondary structures were generated in all 16 groups and were mainly composed of two loops, a large loop and a small loop scattered at the ends of the stem (Fig 1). The length of the stem region was composed of five pairs of bases (Table 2). In addition, free bases were found in groups 4 and 5 of the CRISPR1 locus and group 8 of the CRISPR2 locus, corresponding to MFEs of -15.20 kcal/mol, -15.60 kcal/mol and -15.20 kcal/mol, respectively (Table 2).

**Table 2:** Typical DRs of CRISPR1 and CRISPR2 loci with MFE in nontyphoidal *Salmonella*.

CRISPR loci	Groups	Sequence	MFE	Length (bp)
1	1	CGGTTTATCCCCGCTAGCGCGGGGAACAC	-14.20 kcal/mol	29
1	2	CGGTTTATCCCCGCTGGCGCGGGGAACA	-14.20 kcal/mol	28
1	3	CGGTTTATCCCCGCTGGCGCGGGGAACAC	-14.20 kcal/mol	29
1	4	GTGTTCCCCGCGCCAGCGGGGATAAACCG	-15.20 kcal/mol	29
1	5	GTGTTCCCCGCGCCAGCGGGGATAAACTG	-15.60 kcal/mol	29
1	6	GTTTATCCCCGCTAGCGCGGGGAACAC	-14.20 kcal/mol	27
1	7	GTTTATCCCCGCTGGCGCGGGGAACAC	-14.20 kcal/mol	27
2	1	CGGTTTATCCCCGCTGGCGCGGGGAACA	-14.20 kcal/mol	28
2	2	CGGTTTATCCCCGCTGGCGCGGGGAACAC	-14.20 kcal/mol	29
2	3	CGGTTTATCCCCGCTGGCGCGGGGAACACA	-14.20 kcal/mol	30
2	4	CGGTTTATCCCCGCTGGCGCGGGGAATAC	-14.20 kcal/mol	29
2	5	GGTTTATCCCCGCTGGCGCGGGGAACAC	-14.20 kcal/mol	28
2	6	GTATTCCCCGCGCCAGCGGGGATAAACCG	-15.10 kcal/mol	29
2	7	GTGTTCCCCGCGCCAGCGGGGATAA	-15.10 kcal/mol	25
2	8	GTGTTCCCCGCGCCAGCGGGGATAAACCG	-15.20 kcal/mol	29
2	9	GTTTATCCCCGCTGGCGCGGGGAACAC	-14.20 kcal/mol	27

### CRISPR genotypes and genetic diversity of different nontyphoidal *Salmonella* serogroups

#### CRISPR genotypes and genetic diversity of nontyphoidal *Salmonella* serogroup B

The 161 nontyphoidal *Salmonella* strains belonged to serogroup B (Table 1), including eight serotypes, of which 130 strains were *S*. Typhimurium and its monophasic variant. One was an unidentified serotype. Fifty-five spacer sequences were newly identified, named Unknown 1–28 and Unknown 56–83 (S1 Table), and two new CRISPR genotypes were found.

**Fig 2:**
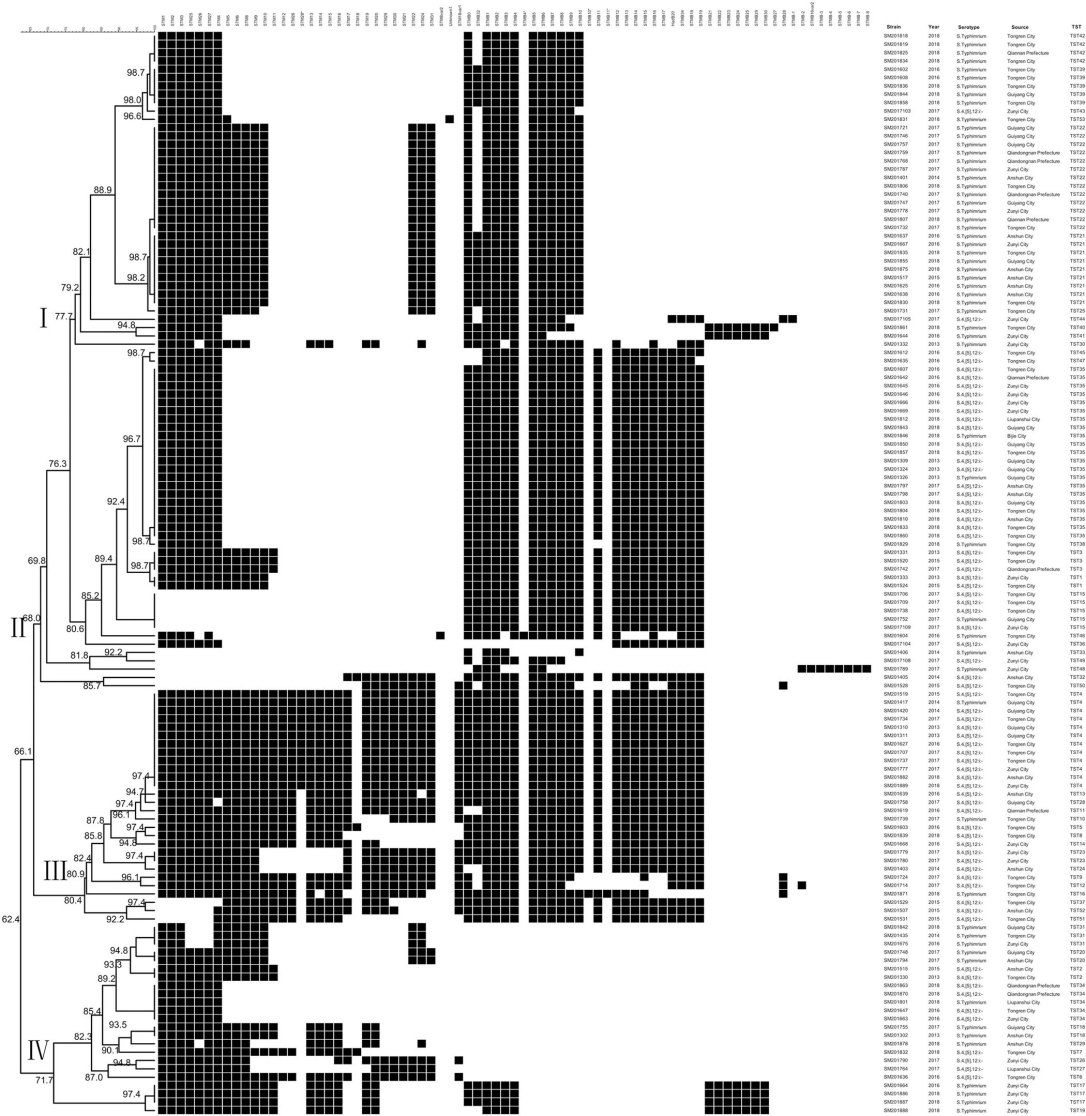
The genetic evolutionary relationship based on the spacer arrangements of 130 *S*. Typhimurium and its monophasic variants strains were analyzed using BioNumerics 8.0 software. The information on nontyphoidal *Salmonella* strains, including strain number, year, isolation location, serotypes, and CRISPR genotypes, was marked on the right. Each black rectangle indicated the presence of the spacers in the strain, and the blank indicated the absence of spacers.

Combining the spacer sequences of CRISPR1 and CRISPR2 loci, 130 strains of *S*. Typhimurium and its monophasic variants were classified into 53 TST genotypes, and a new spacer sequence (unknown1) was identified. Three spacer sequences were duplicative in STM28 of the CRISPR1 locus, STMB10 and STMB11 of the CRISPR2 locus, respectively (Fig 2). While point mutations of the spacer sequences were detected in STM8var2, STM18var1, and STM16var2. For the genotype TST22, one spacer sequence (STMB3) was absent compared to TST21. For the genotype TST24, two spacer sequences (STMB0 and STMB32) were absent compared to TST23. A phylogenic tree based on the spacer sequences of 130 strains were constructed using BioNumerics 8.0 software (Fig 2). The result showed that four genotypes, including TST36, TST15, TST4, and TST34, contained both serotypes of *S*. Typhimurium and its monophasic variants. The remaining TST genotypes were composed of single serotype. The 130 strains of *S*. Typhimurium and its monophasic variants were divided into lineage I-Ⅳ with a genetic similarity of 62.4%-100%. The spacer sequence arrangements of strains revealed a close genetic relationship between *S*. Typhimurium and its monophasic variants.

**Fig 3:**
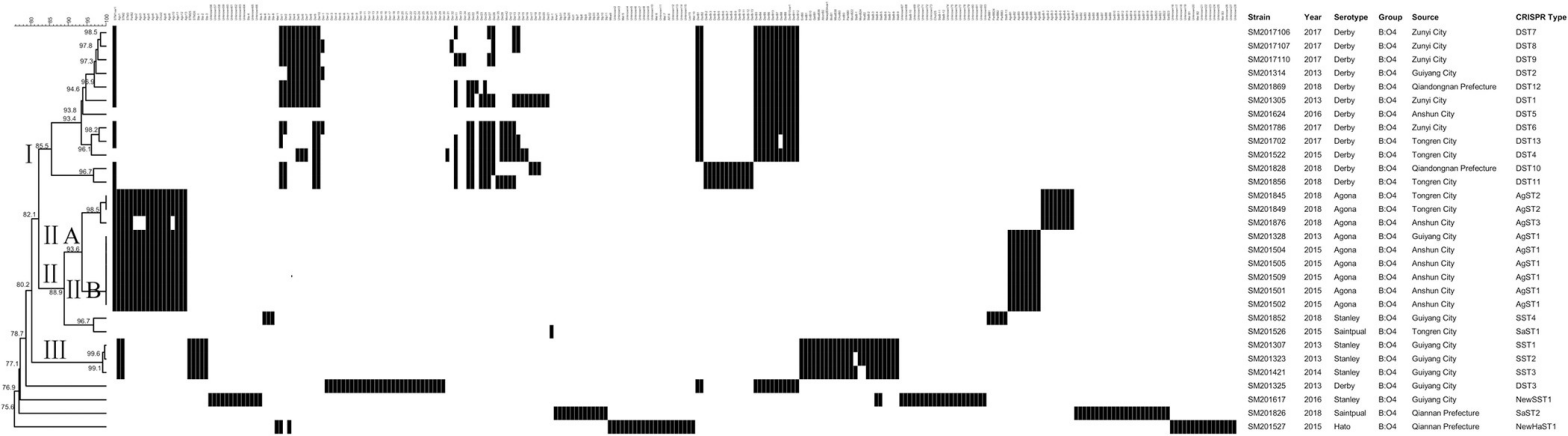
The genetic evolutionary relationship based on the spacer arrangements of 30 nontyphoidal *Salmonella* strains belonging to serogroup B was analyzed using BioNumerics 8.0 software. The information on nontyphoidal *Salmonella* strains, including strain number, year, isolation location, serotypes, and CRISPR genotypes, was marked on the right. Each black rectangle indicated the presence of the spacers in the strains, and the blank indicated the absence of spacers. A "*" in the spacer arrangement indicated a duplicate spacer, and a "var" indicated a point mutation in the spacer.

Except for *S*. Typhimurium and its monophasic variants, the other 30 *Salmonella* serogroup B strains were classified into 24 CRISPR genotypes with 54 new spacer sequences and two new CRISPR genotypes. The spacer sequence arrangements of 30 strains were clustered into three lineages with a genetic similarity of 75.6–100% (Fig 3). Twelve distinct DST genotypes of 12 *S*. Derby strains were gathered in lineage I with a genetic similarity of 85.5–98.5%. Nine *S*. Agona strains were gathered in lineage ⅡA with a genetic similarity of 93.6–100%. Among these strains, six *S*. Agona strains were identical AgST1 genotype, one from Guiyang city in 2013 and five from Anshun city in 2015. Two new CRISPR genotypes were NewSST1 of *S*. Stanley and NewHaST1 of *S*. Hato, respectively. The spacers of AgST1 and AgST2 were identical at CRISPR1 locus but different at CRISPR2 locus. However, the spacer of AgST3 and AgST2 were identical at CRISPR2 loci but different at CRISPR1 locus. Four spacers (Ago2, Ago3, Ago4, and Ago10) of AgST3 at CRISPR1 locus were absent.

#### CRISPR genotypes and genetic diversity of nontyphoidal *Salmonella* serogroup C1

**Fig 4:**
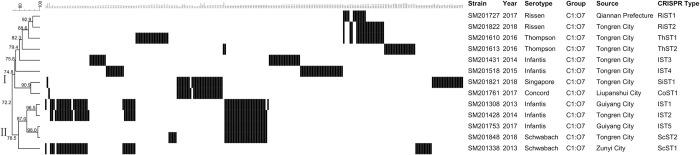
The genetic evolutionary relationship and spacer arrangements of 13 nontyphoidal *Salmonella* strains belonging to serogroup C1 were analyzed using BioNumerics 8.0 software. The information on nontyphoidal *Salmonella* strains, including strain number, year, isolation location, serotypes, and CRISPR genotypes, was marked on the right. Each black rectangle indicated the presence of the spacers in the strain, and the blank indicated the absence of spacers.

Thirteen nontyphoidal *Salmonella* strains belonged to serogroup C1 with seven serotypes, which were divided into 13 different CRISPR genotypes and two lineages with a genetic similarity of 72.2% (Fig 4). Five *S*. Infantis strains were divided into five different CRISPR genotypes and distributed in two clusters. The two *S*. Infantis strains (SM201428, SM201753) and one *S*. Schwabach strain (SM201848) shared the same spacer sequence at the CRISPR2 locus with a genetic similarity of 98.0%. One *S*. Singapore strain (SM201821) and one *S*. Concord strain (SM201761) shared a part of the spacer sequences at the CRISPR1 locus with a genetic similarity of 90.9%. one *S*. Rissen strain (SM201822) and one *S*. Thompson strain (SM201610) were identical at the CRISPR2 locus with a genetic similarity of 88.6%.

#### CRISPR genotypes and genetic diversity of nontyphoidal *Salmonella* serogroup C2-C3

**Fig 5:**
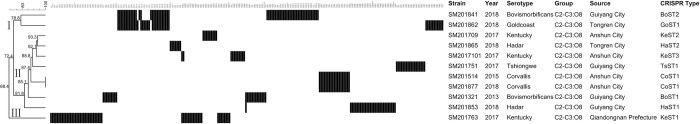
The genetic evolutionary relationship and spacer arrangements of 11 nontyphoidal *Salmonella* strains belonging to serogroup C2-C3 were analyzed using BioNumerics 8.0 software. The information on nontyphoidal *Salmonella* strains, including strain number, year, isolation location, serotypes, and CRISPR genotypes, was marked on the right. Each black rectangle indicated the presence of the spacers in the strain, and the blank indicated the absence of spacers.

Eleven nontyphoidal *Salmonella* strains belonged to serogroup C2-C3 with seven serotypes, which were classified into 10 CRISPR genotypes and three lineages with a genetic similarity of 68.4% (Fig 5). One *S*. Bovismorbifican strain and one *S*. Goldcoast strain were in lineages I, which shared identical spacers at the CRISPR1 locus with a genetic similarity of 78.8%. In lineage II, six CRISPR genotypes were detected in seven nontyphoidal *Salmonella* strains, of which two *S*. Corvallis strains were classified as an identical CRISPR genotype (CoST1). Only one *S*. Kentucky strain was in lineage III.

#### CRISPR genotypes and genetic diversity of nontyphoidal *Salmonella* serogroup D1

**Fig 6:**
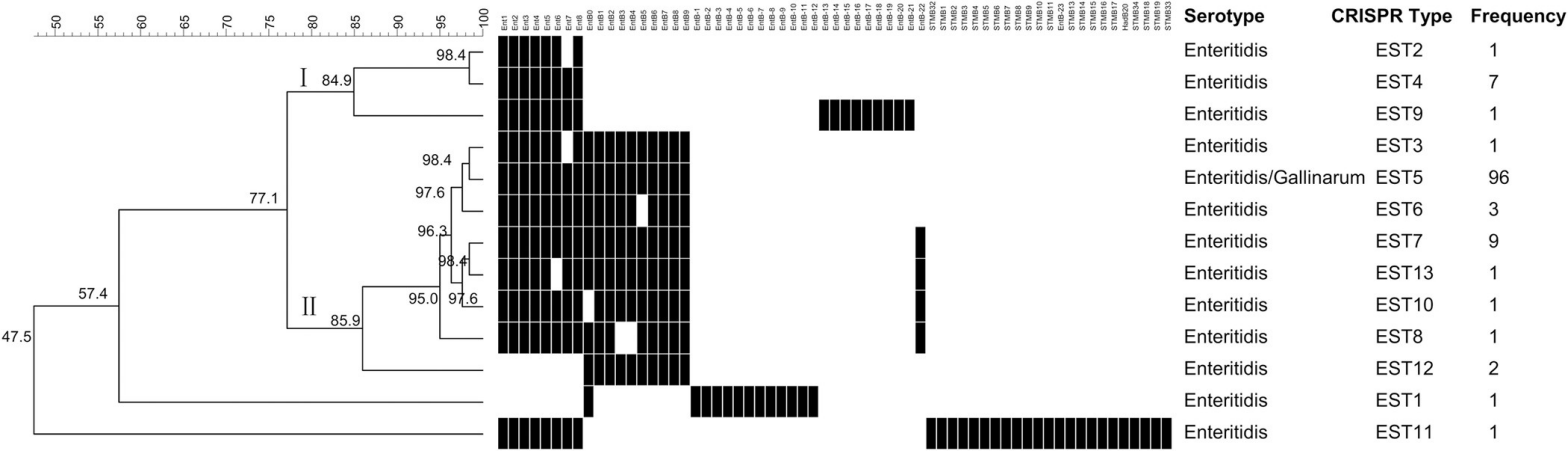
The genetic evolutionary relationship and spacer arrangements of 124 *S*. Enteritidis strains and one *S*. Gallinarum strain belonging to serogroup D1 were analyzed using BioNumerics 8.0 software. The information on nontyphoidal *Salmonella* strains, including serotype, CRISPR type, and frequency of CRISPR type, was marked on the right. Each black rectangle indicated the presence of the spacers in the strains, and the blank indicated the absence of spacers.

One hundred twenty-five *Salmonella* strains belonging to serogroup D1 were classified into 13 different CRISPR genotypes with a genetic similarity of 47.5% (Fig 6). Two serotypes were detected, including 124 *S*. Enteritidis strains and one *S*. Gallinarum strain. 76.8% of strains were divided into the same EST5 genotype including one *S*. Gallinarum strain. In addition, the EST5 genotype had one more spacer than EST3, EST6, EST13, and EST10, respectively, with a genetic similarity of more than 95.0%. Meanwhile, EST5 had two more spacers (EntB2 and EntB3) than EST8. Compared with the spacers of EST4, the spacer of Ent7 was absent in EST2. Only one *S*. Enteritidis strain was identified in each of the eight genotypes, including EST1, EST2, EST3, EST8, EST9, EST10, EST11, and EST13.

#### CRISPR genotypes and genetic diversity of nontyphoidal *Salmonella* serogroup E1

**Fig 7:**
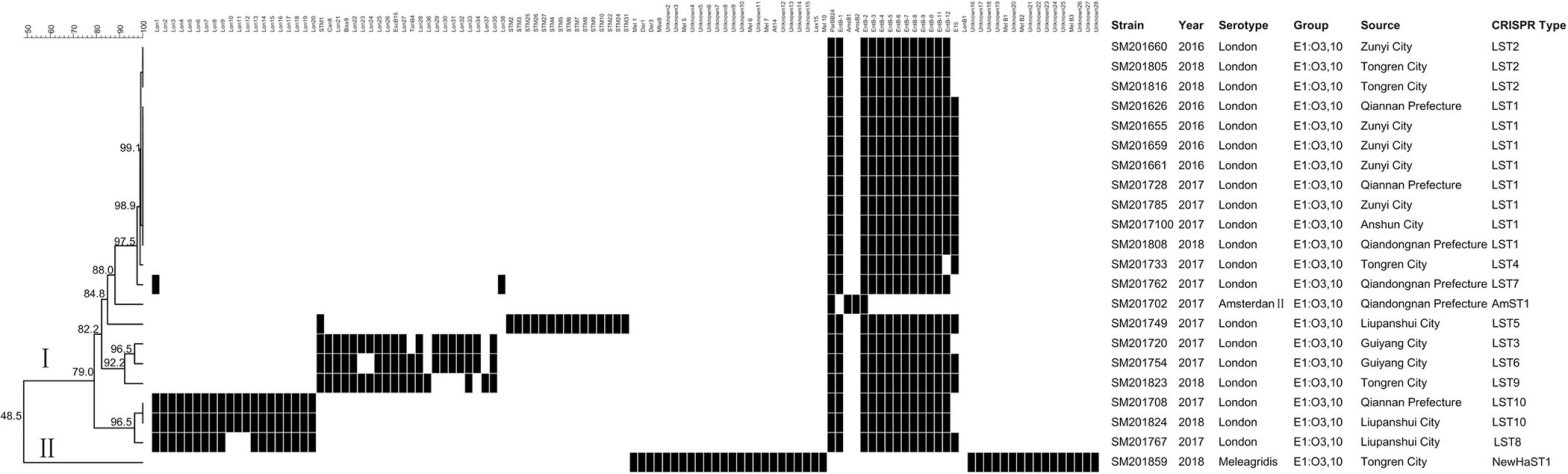
The genetic evolutionary relationship and spacer arrangements of 22 strains belonging to serogroup E1 were analyzed using BioNumerics 8.0 software. The information on nontyphoidal *Salmonella* strains, including strain number, year, isolation location, serotypes, and CRISPR genotypes, was marked on the right. Each black rectangle indicated the presence of the spacers in the strain, and the blank indicated the absence of spacers. For the new spacer sequence not reported in the known spacer database, we named them unknown2-28.

Twenty-two nontyphoidal *Salmonella* strains belonged to serogroup E1 including three serotypes, which were classified into 12 distinct CRISPR genotypes and two lineages with a genetic similarity of 48.5% (Fig 7). Twenty-seven new spacers (unknown2-28) and one new CRISPR genotype (NewHaST1) were found. Interestingly, one *S*. Meleagridis strain had identical spacer arrangements with one *S*. Hato strain belonging to the serogroup B. Twenty-one *Salmonella* strains, including twenty *S*. London strains and one *S*. Amsterdam Ⅱ strain, were clustered in lineage I with a genetic similarity of 79%. Three genotypes (LST1, LST2, and LST4) were not detected spacer sequences at CRISPR1 locus. However, three spacers, including LonB12, LonB13, and LonB14, were different at CRISPR2 locus with a closer genetic similarity of 97.5%.

#### CRISPR genotypes and genetic diversity of unidentified nontyphoidal *Salmonella* serotypes

A total of 53 novel spacers (unknown 29–55, unknown 84–108) were identified in 11 nontyphoidal *Salmonella* strains with unknown serotypes. Moreover, five novel CRISPR genotypes were found in the serogroups E1 and C2-C3 and distributed in 2013 and 2014 (Table 3). Four novel CRISPR genotypes were identified in the serogroup E1 and showed identical or shareable spacers with a close genetic similarity. The spacer of Unknown42 was absent at the CRISPR1 locus in Newtype4 compared with the Newtype1, Newtype2, and Newtype3. In addition, the spacers of Unknown 46 and Unknown 47 were absent at the CRISPR2 locus in Newtype4 compared to Newtype2 and Newtype3. The same spacer arrangement was found at the CRISPR1 locus in Newtype3 and Newtype2, while a new spacer of Unknown55 was identified at the CRISPR2 locus in Newtype3.

**Table 3:** Spacers and CRISPR genotypes of 11 unidentified nontyphoidal *Salmonella* strains.

No. Strain	Year	Serogroup	CRISPR genotype	CRISPR loci	CRISPR spacer
**SM201628**	2016	B	Type1	CRISPR1	Ind1+Had12+Had13+Ind2+Ind3+Ind4+Ind14+Ind15+Ind5+Ind6+Ind7+Ind8+Ind9+Ind10+Ind11+Ind12+Ind13
				CRISPR2	EntB0+IndB1+GallB2+CholB1+CholB2+CholB3+IndB3+IndB4+IndB5+IndB6+IndB7+IndB8+IndB9+IndB10+IndB11+IndB12+IndB13
**SM201316**	2013	E1	Newtype1	CRISPR1	E1+E2+Unknown29+Unknown30+Unknown31+Unknown32+E3+E4+Unknown33+E5+Unknown34+Unknown35+Unknown36+Unknown37+Unknown38+Unknown39+Unknown40+E6+Unknown41+Unknown42+E7+E8
				CRISPR2	Unknown17+Unknown18+Unknown19+MelB1+Unknown20+MelB2+Unknown21+Unknown22+Unknown23+Unknown24+Unknown25+MelB3+Unknown26+Unknown27+Unknown28
**SM201317**	2013	E1	Type2	CRISPR1	Ent1+Ent2+Ent3+Ent4+Ent5+Ent6+Ent7+Ent8
				CRISPR2	
**SM201318**	2013	E1	Newtype2	CRISPR1	E1+E2+Unknown29+Unknown30+Unknown31+Unknown32+E3+E4+Unknown33+E5+Unknown34+Unknown35+Unknown36+Unknown37+Unknown38+Unknown39+Unknown40+E6+Unknown41+Unknown42+E7+E8+E12+E13
				CRISPR2	Unknown49+Unknown43+Unknown44+E9+Unknown45+Unknown46+Unknown47+Unknown48+Unknown54+E10+Unknown50+E11+Unknown51+Unknown52+Unknown53
**SM201319**	2013	E1	Newtype2	CRISPR1	E1+E2+Unknown29+Unknown30+Unknown31+Unknown32+E3+E4+Unknown33+E5+Unknown34+Unknown35+Unknown36+Unknown37+Unknown38+Unknown39+Unknown40+E6+Unknown41+Unknown42+E7+E8+E12+E13
				CRISPR2	Unknown49+Unknown43+Unknown44+E9+Unknown45+Unknown46+Unknown47+Unknown48+Unknown54+E10+Unknown50+E11+Unknown51+Unknown52+Unknown53
**SM201423**	2014	E1	Newtype3	CRISPR1	E1+E2+Unknown29+Unknown30+Unknown31+Unknown32+E3+E4+Unknown33+E5+Unknown34+Unknown35+Unknown36+Unknown37+Unknown38+Unknown39+Unknown40+E6+Unknown41+Unknown42+E7+E8+E12+E13
				CRISPR2	Unknown49+Unknown43+Unknown44+E9+Unknown45+Unknown46+Unknown47+Unknown48+Unknown54+E10+Unknown50+E11+Unknown51+Unknown52+Unknown53+**Unknown55**
**SM201425**	2014	E1	Type3	CRISPR1	
				CRISPR2	ParBB24+EntB-1+EntB-2+EntB-3+EntB-4+EntB-5+EntB-6+EntB-7+EntB-8+EntB-9+EntB-10+EntB-11+E14+E15
**SM201412**	2014	E1	Type4	CRISPR1	Ent1+Ent2+Ent3+Ent4+Ent5+Ent6+Ent7+Ent8
				CRISPR2	E16+E17+E18+E19
**SM201429**	2014	E1	Newtype4	CRISPR1	E1+E2+Unknown29+Unknown30+Unknown31+Unknown32+E3+E4+Unknown33+E5+Unknown34+Unknown35+Unknown36+Unknown37+Unknown38+Unknown39+Unknown40+E6+Unknown41+E7+E8+E12+E13
				CRISPR2	Unknown49+Unknown43+Unknown44+E9+Unknown45+Unknown48+Unknown54+E10+Unknown50+E11+Unknown51+Unknown52+Unknown53+Unknown55
**SM201634**	2016	C1	Type5	CRISPR1	STM1var3+C1+C2+C3+C4+C5+C6+C7+C8+C 9+C10+C11+C12+C13+C14+C15+C16+C17+C18+C19+C20
				CRISPR2	CB1+CB2+CB3+CB4+CB5+CB6+CB7
**SM201426**	2014	C2-C3	Newtype5	CRISPR1	
				CRISPR2	InfB2+Unknown84+Unknown85+Unknown86+Unknown87+CB8+Unknown88+CB9+Unknown89+CB10+Unknown90+CB11+Unknown91+Unknown92+Unknown93+Unknown94+Unknown95+Unknown96+CB12+Unknown97+Unknown98+Unknown99+CB13+Unknown100+Unknown101+Unknown102+Unknown103+Unknown104+CB14+Unknown105+Unknown106+Unknown107+Unknown108

## Discussion

Nontyphoidal *Salmonell*a was one of the most important bacterial pathogens associated with a large number of foodborne disease outbreaks [2,23]. Salmonellosis has become a significant public health problem worldwide, causing approximately 115 million infections and 370,000 deaths globally [24]. From 2009–2018, The national-based prospective surveillance of all-ages patients with acute diarrhea showed that *Escherichia coli* and nontyphoidal *Salmonella* were the two leading bacterial pathogens in China [25]. Nontyphoidal *Salmonella* usually causes self-limiting gastroenteritis in humans and animals, while a few nontyphoidal *Salmonella* can also cause invasive nontyphoidal *Salmonella* (iNTS) life-threatened [26]. Until now, more than 50 serogroups have been identified [4]. Serogroups B and D were the most common nontyphoidal *Salmonella* serogroups worldwide, while serogroups C1 and C2-C3 were also highly associated with human and animal health [27]. In this study, nontyphoidal *Salmonella* serogroup B was the dominant serogroup, followed by serogroup D1. The serogroup prevalence observed in Guizhou was similar to that reported in other cities in China [28,29]. However, nontyphoidal *Salmonella* serogroup C was the dominant serogroup in the United States [4]. Meanwhile, *S*. Typhimurium monophasic variant has gradually become the leading prevalent serotype in recent years [30,31]. In addition, it has surpassed *S*. Enteritidis and *S*. Typhimurium as the predominant serotype in human isolates [32–34]. The prevalent serogroups and serovars of nontyphoid *Salmonella* were diverse in different regions and years. Therefore, monitoring the dynamic change of nontyphoidal *Salmonella* was essential.

Subtyping nontyphoidal *Salmonella* is necessary for microbiological identification, epidemiological investigation, and outbreak tracking. With the rapid development of molecular biology, there are various methods for *Salmonella* subtyping. CRISPR is one of the subtyping methods [13,15,35]. The RNA secondary structure of typical DRs were predicted using RNAfold and CRISPRCasFinder, and DRs and spacer sequences were displayed. Directed Repeats had palindromic structures and could be transcribed into RNA secondary structures [36]. The stem-loop structure of typical DRs may facilitate the recognition of exogenous DNA or RNA with Cas proteins [18]. In our study, 16 groups of typical DRs could form stable RNA secondary structures in 342 nontyphoidal *Salmonella* strains, including seven groups at CRISPR1 loci and nine groups at CRISR2 loci. In this study, the stability of RNA secondary structure was positive correlation with the DRs length and GC content, which was the same as previous studies [37]. However, free bases were found in groups 4 and 5 of the CRISPR1 locus and group 8 of the CRISPR2 locus, corresponding to MFEs of -15.20 kcal/mol, -15.60 kcal/mol and -15.20 kcal/mol, respectively. The MFEs were higher than those of other groups, and whether they affected the stability of RNA secondary structure need to be further investigated.

The DRs of CRISPR locus were relatively conserved [38]. However, the spacer sequences were different in different pathogens or different serotypes of the same pathogen. The acquisition, loss, or duplication of spacer sequences resulted in one of the fastest evolutionary loci in bacteria. Therefore, the specificity of spacer sequences can accurately show the relationship and epidemiology between different strains from different environments and locations [39]. Our study found the presence of spacer sequences with duplication or mutation in *S*. Typhimurium and its monophasic variants strains. Three spacer sequences were duplicative in STM28 of the CRISPR1 locus, STMB10 and STMB11 of the CRISPR2 locus, respectively. While point mutations of the spacer sequences were detected in STM8var2, STM18var1, and STM16var2. Deletion or acquisition of some spacer sequences in CRISPR1 or CRISPR2 loci gave rise to new CRISPR genotypes. For example, the spacer of STMB3 was absent in TST22 compared to TST21, and the spacers of STMB0 and STMB32 were absent in TST24 compared to TST22. In *S*. Enteritidis strains, the spacer of Ent10 was identified in EST7 compared to EST10. In contrast, the spacer of Ent10 of the EST5 genotype was absent in EST3. Three strains (SM201318, SM201319 and SM201423) of unidentified nontyphoidal *Salmonella* serotypes were identified two new CRISPR genotypes, newtype2 and newtype3, due to having an unknown55 spacer at the CRISPR2 locus. In addition, AgST3 was absent from four spacers compared to AgST2 at the CRISPR1 loci with a genetic similarity of 98.5%. There were only two spacer differences between LST2, LST1, and LST4, with a genetic similarity of 97.5%. These results indicated that nontyphoidal *Salmonella* was constantly evolving. The diversity of nontyphoidal *Salmonella* strains was not only the deletion, duplication, or point mutation of spacer sequences but also the acquisition of new spacer sequences to form novel genotypes, which was similar to the previous reports [19,40,41].

The 342 nontyphoidal *Salmonella* strains were divided into 135 CRISPR genotypes in this study. The emerging *S*. Typhimurium monophasic variants were considered as one of the derivatives of *S*. Typhimurium with close antigenic formula and genetic relationship, which have been significantly increasing in prevalence worldwide [30,42]. In our study, 130 strains of *S*. Typhimurium and its monophasic variants were classified into 53 CRISPR genotypes. The correlation between CRISPR genotypes and serotypes revealed that CRISPR genotyping could distinguish between *S*. Typhimurium and its monophasic variants. At the same time, we noticed that 124 *S*. Enteritidis strains were divided into 13 CRISPR genotypes, among which eight CRISPR genotypes had only one strain. The result showed that CRISPR genotyping had a lower distinguish power for *S*. Enteritidis, the same as the previous study in Jiangsu, China [13]. Interestingly, we discovered that the only one *S*. Gallinarum strain was identical spacer arrangements with EST5 of *S*. Enteritidis, which should be further investigated for the possible reason.

In our study, the CRISPR genotyping based on the spacer sequence was an effective method that can reveal the genetic diversity of nontyphoidal *Salmonella* serogroup B, serogroup C1, serogroup C2-C3, and serogroup E1. For 11 *Salmonella* strains of unknown serotypes, 28 new spacers were identified in serogroup E1. Meanwhile, five new CRISPR genotypes were discovered based on new spacers at CRISPR1 and CRISPR2 loci. The genotypes of these strains differed from each other due to a spacer sequence with a close similarity. The spacer sequences of CRISPR were closely related to *Salmonella* serotypes [20]. Therefore, we speculated that those strains of new CRISPR genotypes shared the same serotype, which needs further to be confirmed by the whole genome sequence (WGS) of strains.

In summary, we described the RNA secondary structure of typical direct repeats (DRs), spacer sequences, genotypes, and genetic diversity of nontyphoidal *Salmonella* strains from Guizhou using CRISPR genotyping. This study indicated that nontyphoidal *Salmonella* was constantly evolving. The diversity of nontyphoidal *Salmonella* CRISPR loci was not only the deletion, duplication, or point mutation of spacer sequences but also the acquisition of new spacer sequences to form novel genotypes. The CRISPR genotyping based on the spacer sequence effectively reveals the genetic diversity of nontyphoidal *Salmonella* serogroup B, serogroup C1, serogroup C2-C3, and serogroup E1 except for *S*. Enteritidis.

## Supporting information

S1 Table 981 spacer sequences of CRISPR loci for 342 nontyphoidal *Salmonella* strains from Guizhou province, China. (XLSX)
